# Deep sequencing shows that accumulation of potentially pathogenic mtDNA mutations rather than mtDNA copy numbers may be associated with early embryonic loss

**DOI:** 10.1007/s10815-020-01893-5

**Published:** 2020-07-23

**Authors:** Licheng Ji, Tingting Liao, Juan Yang, Houming Su, Jianyuan Song, Kun Qian

**Affiliations:** 1 Reproductive Medicine Center, Tongji Hospital, Tongji Medical College, Huazhong University of Science and Technology, 1095 JieFang Avenue, Wuhan, 430030 Hubei China; 2 The Fourth Affiliated Hospital, Zhejiang University School of Medicine, No.1 Shang Cheng Avenue, Yiwu, Zhejiang, 322000 China

**Keywords:** Spontaneous abortion, Mitochondria, mtDNA copy number, Heteroplasmy, Next-generation sequencing (NGS)

## Abstract

**Purpose:**

To explore the relationship between mitochondrial DNA quantity and heteroplasmy and early embryonic loss.

**Methods:**

A total of 150 villous samples from patients with spontaneous abortion (SA, *n* = 75) or induced abortion (IA, *n* = 75) were collected. qPCR and next-generation sequencing (NGS) were used to test mitochondrial DNA quantity and heteroplasmy. Missense mutations with a CADD score > 15 and heteroplasmy ≥ 70% were defined as potentially pathogenic mutations.

**Results:**

With respect to mitochondrial DNA copy numbers, there was no significant difference between the SA and IA groups (median (IQR), 566 (397–791) vs. 614 (457–739); *P* = 0.768) or between the euploid and aneuploid groups (median (IQR), 516 (345–730) vs. 599 (423–839); *P* = 0.107). mtDNA copy numbers were not associated with spontaneous abortion using logistic regression analysis (*P* = 0.196, 95% CI 1.000–1.001). In addition, more patients harbored possibly pathogenic mtDNA mutations in their chorionic villi in the SA group (70.7%, 53/75) compared with the IA group (54.7%, 41/75; *P* < 0.05). However, there was no statistical difference between the euploid (80%, 24/30) and aneuploid groups (64.4%, 29/45; p = 0.147).

**Conclusion:**

Early embryonic loss and the formation of aneuploidy were not related to mtDNA copy number. Patients with spontaneous abortion were more likely to have possibly pathogenic mutations in their mtDNA, and this may assist in purifying pathogenic mtDNA. However, whether the accumulation of these potentially morbific mtDNA mutations caused early embryonic loss requires further investigation.

## Introduction

About 10–15% of clinically recognized pregnancies result in miscarriage, with aneuploidy as the principal cause [1]. Trisomies are the most frequently observed type of aneuploidy (61.2%), followed by triploidies (12.4%), monosomy X (10.5%), tetraploidies (9.2%), and structural chromosomal anomalies (4.7%) [2].

In human cells, however, there is another genome—the mitochondrial (mtDNA) genome. In contrast to only two copies of nuclear DNA, a single human cell hosts hundreds to thousands of copies of mtDNA; the mitochondrially encoded proteins are essential for mitochondrial oxidative phosphorylation and cellular energetics [3].

mtDNA mutations often only affect a small proportion of a cell’s mtDNA (a state termed heteroplasmy), which determines the integrity, or “quality” of the mtDNA [4]. The number of mtDNA molecules or “mtDNA copy number” is also strictly regulated, ensuring that mitochondria can generate appropriate levels of energy and intracellular signals to maintain normal cellular functions [5].

mtDNA does not replicate in the period between when the oocyte is in MII and when embryonic hatching occurs except in response to respiratory chain defect [6] or after cryopreservation procedures [7], and mitochondria are randomly assigned to each blastomere. Early embryonic development thus depends entirely on the storage of mitochondria in oocytes to provide energy [8], and the number and quality of mitochondria are determinants of the developmental potential of oocytes and early embryos [9], In this paper, we therefore evaluated mitochondrial DNA quantity and quality by a deep sequencing technique and qPCR of the chorionic villi procured from human first trimester miscarriages that were either euploid or aneuploid.

## Materias and methods

### Subjects

We obtained approval from the Ethics Committee of Tongji Hospital, Huazhong University of Science and Technology. No conflicts of interest were declared. One hundred fifty villous samples were collected by curettage or manual vacuum aspiration at Tongji Hospital between January 2019 and July 2019 and a written consent was obtained from each patients*.* Fetal cardiac activity and gestational age were examined by ultrasonography 6 weeks after the patient’s last menstrual period. All first trimester placentas (6–10 weeks gestation) were obtained from women aged 20 to 41. The spontaneous abortion (SA) group consisted of 75 women with either a fetal sac without any heart activity, a fetal sac with a maximal diameter greater than 11 mm without the yolk sac, or an empty fetal sac where the gestational age was confirmed at not less than 6 weeks [10]. All of the pregnancies were conceived naturally. We performed single-nucleotide polymorphism arrays in the SA group to obtain the CNV (copy-number variant) information. The induced abortion (IA) group consisted of 75 women with normal fetal heart activity who did not wish to continue the pregnancy. To avoid maternal contamination, the samples collected were washed repeatedly to remove maternal blood cells and impurities. The clean villi were then collected under the anatomical microscope. And STR techniques was used to determine whether there is maternal contamination in the specimen. Each sample was stored at − 80 °C until DNA extraction.

### Extraction of DNA

Total DNA was extracted from villous samples using the QIAamp DNA tissue Mini Kit (Qiagen, Hilden, Germany) according to the manufacturer’s instructions. The pure genomic DNA was 50 ng of DNA per μl of the buffer, and the ratio of absorbance at A260/280 was required to be near 1.8 OD units.

### Quantification of mtDNA copy numbers

To determine the mtDNA copy number, we performed real-time quantitative PCR (qPCR) using a Master Mix System (Roche®, USA). The primers selected were tRNA-Leu-F (forward, 5′-CACCCAAGAACAGGGTTTGT-3′ and reverse, 5′-TGGCCATGGGTATGTTGTTA-3′) to quantify the mtDNA; beta-actin (B2-microglobulin; forward, 5′-TGCTGTCTCCATGTTTGATGTATCT-3′ and reverse, 5′-TCTCTGCTCCCCACCTCTAAGT-3′) to quantify the nuclear DNA; and GAPDH F/R (forward, 5′-AGAAGGCTGGGGCTCATT-3′ and reverse, 5′-TGCTAAGCAGTTGGTGGTG-3′) as a housekeeping gene.

We obtained CT (raw threshold cycle) values from real-time PCR software and averaged the CT values from triplicate reactions. To determine the mitochondrial DNA content relative to nuclear DNA, we used the following equations: (a) ΔCT B2 = (CT B2 –CT GAPDH) ΔCT tRNA-Leu = (CT tRNA-Leu –CT GAPDH) and (b) relative mitochondrial DNA content = 2^CTB2-CTtRNA-Leu^ [11].

### mtDNA amplification and sequencing

Multiplex PCR was used to amplify mtDNA. We prepared sequencing libraries using a MultipSeq AImumiCap Panel (iGene Tech), and indexed libraries were multiplexed and run on an Agilent 2100 Bioanalyzer system (Agilent DNA 1000 Kit) that generated 220–320-bp fragments. The input library pool concentration was 1–10 ng/μl, and libraries were sequenced with the Illumina HiSeq Xten sequencing system (Illumina, Inc., San Diego, CA, USA).

Sequenced original data files were converted into original-sequencing sequences (based sequence analysis) by base calling analysis, and the results were stored in the FASTQ file format. In order to ensure the quality of the information analysis, preliminary reads needed to be filtered to obtain clean reads without adapter information, low-quality bases, or undetected bases. The alignment software BWA (Burrows-Wheeler Aligner) 0.7.15 was used to compare the high-quality sequencing data to the human mitochondrial reference genome (NC_012920.1) to obtain the comparison results file BAM. We used Samtools (1.5) to calculate mitochondrial sequencing depth and coverage from the aligned BAM files. VarScan (version 2.3.9) was used to analyze SNP and Indel mutation and to retain mutations with mutation frequencies greater than 1%. SnpEff (4.3) software was used to annotate all mutations in genes and functional intervals and limit its upstream interval to 2 k. We used SnpSift to annotate mutations in the 1000 Genomes Project, Mitomap, ClinVar, etc.

The Combined Annotation-Dependent Depletion (CADD, https://cadd.gs.washington.edu/) score integrated many different annotations of the variant (including functionality, pathogenicity, and experimentally measured effects) into one score, which was used to predict the pathogenic potential of variants. A CADD score of 15 points was suggested to define a pathogenic mutation [12].

### CNV gene content

The sequencings from the missed-abortion group reads were mapped to the human genome version 19 (hg19) using the Burrows-Wheeler Aligner. Gene names and chromosomal coordinates for CNVs were mined from the Database of Genomic Variants (DGV, http://dgv.tcag.ca/dgv/app/home), Online Mendelian Inheritance in Man (OMIM, http://www.omim.org), Database of Chromosomal Imbalance and Phenotype in Humans using Ensembl Resources (DECIPHER, http://decipher.sanger.ac.uk/), and UCSC (http://genome.ucsc.edu/).

### Statistical analysis

We performed all statistical analyses using IBM SPSS Statistics for Windows 23.0, GraphPad Prism version 7.0, or R Statistical Software. We used the unpaired Student's *t* test for comparisons of normally distributed data and the Wilcoxon signed-rank test for non-normally distributed data. Correlations were determined using logistic regression analysis. Chi-square test was used to compare the proportion of patients harboring possibly pathogenic mtDNA mutations in different groups. The data are presented as the median ± interquartile range (IQR). P < 0.05 was considered to indicate a statistically significant difference.

## Results

### mtDNA copy numbers in villous samples

Percentage of number of miscarriage was shown in Table 1. Table 2 shows that the age of the patients in the SA group was significantly higher than in the IA group (median, 31 (IQR 28–34) vs. 28 [(IQR, 25–31) years; *P* < 0.001). However, with respect to mitochondrial DNA copy numbers, we observed no significant difference between the SA and IA groups (median, 566 (IQR, 397–791) vs. 614 (IQR, 457–739); *P* = 0.768; Table 2).

**Table 1:** Percentage of number of miscarriage

Number of miscarriage	Overall, *N* = 75	Aneuploid, *N* = 45	Euploid, *N* = 30
1	48.00% (36/75)	46.67% (21/45)	50.00% (15/30)
2	29.33% (22/75)	31.11% (14/45)	26.67% (8/30)
≥ 3	13.33% (10/75)	13.33% (6/45)	13.33% (4/30)

**Table 2:** Maternal age and mtDNA copy numbers in villous samples

	Overall	SA			IA	*P* value
	*N* = 150	Overall *N* = 75	Aneuploid *N* = 45	Euploid *N* = 30	*N* = 75	
Maternal age median (IQR)	29 (26–33)	31 (28–34)	31 (28–34)	30 (28–33)	28 (25–31)	**<** *0.001*
mtDNA copy number median (IQR)	583 (420–760)	566 (397–791)	599 (423–839)	516 (345–730)	614 (457–739)	0.768

*SA*, spontaneous abortion; *IA*, induced abortion; *IQR*, interquartile range; *mtDNA*, mitochondrial DNA The differences in italics were considered significant at P<0.05.

We successfully obtained chromosomal ploidy information for the SA group by CNV analysis. Of the 75 samples, 45 were found to contain an aneuploid karyotype; and the aneuploidy rate was 60%.

**Fig. 1:**
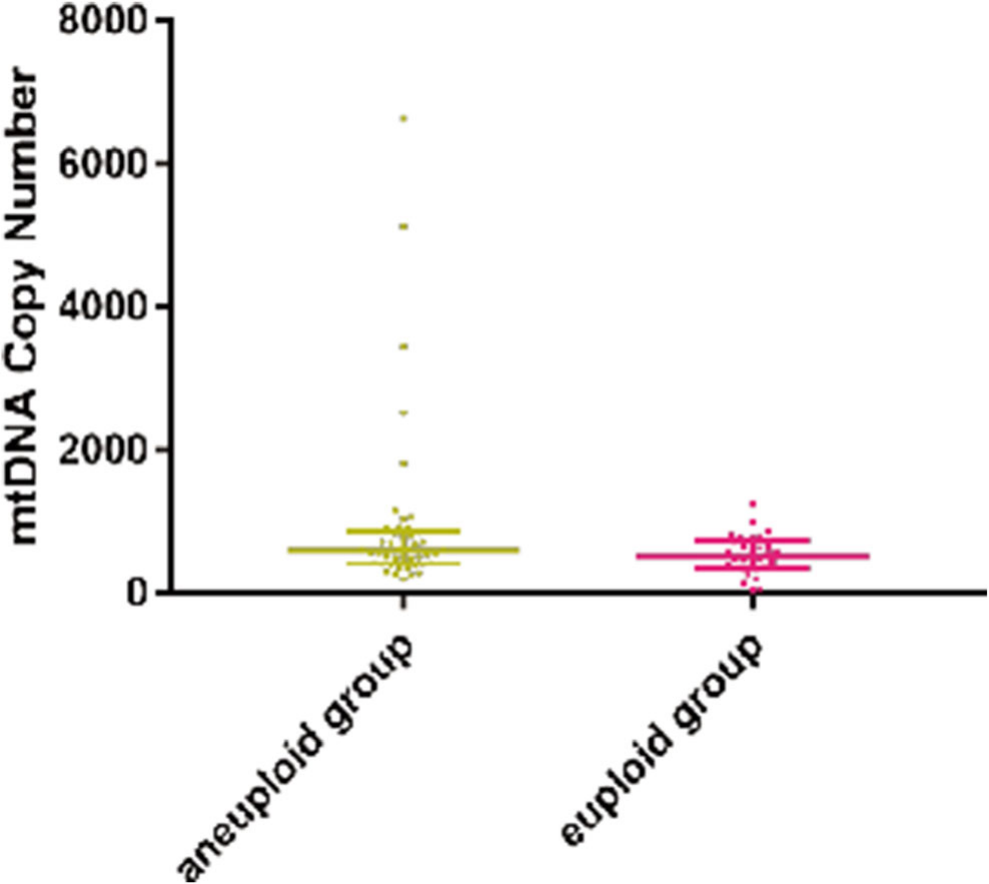
mtDNA copy numbers of patients in the aneuploid and euploid groups. Although we found no statistical difference between them (median (IQR), 516 (345–730) vs. 599 (423–839); *p* = 0.107), the maximal 5 values (6635, 5132, 3445, 2514, and 1820) appeared in the aneuploid group

Next, to explore the relationship between aneuploidy formation and mitochondrial DNA quantity, we compared the mtDNA copy numbers of the aneuploid group with those of the euploid group. As shown in Fig. 1, the median mtDNA copy number of villous samples in the aneuploid group (*n* = 45) was 599 (IQR, 423–839), which showed a higher tendency than that in the euploid group (*n* = 30; median, 516; IQR, 345–730), but this difference was not statistically significant (*P* = 0.107). Moreover, although mtDNA copy number in the IA group (median, 614; IQR, 457–739) showed a higher tendency than that of in the SA euploid group (median, 516; IQR, 345–730), Wilcoxon signed-rank test showed no statistical difference between the two groups (*P* = 0.523). However, it was interesting that 5 patients in the aneuploid group had extremely high mtDNA copy numbers (6635, 5132, 3445, 2514, and 1820). They were all pregnancies with fetal sac without any heart activity.

### Relationships among maternal age, mtDNA copy numbers, and spontaneous abortion

We carried out binary logistic regression analysis to evaluate the effect of mtDNA copy numbers and maternal age on spontaneous abortion (Table 3), and showed that maternal age was an independent risk factor for miscarriage (*P* = 0.001; 95% CI, 1.050–1.210). However, the copy numbers of mtDNA were not associated with spontaneous abortion (*P* = 0.196; 95% CI, 1.000–1.001).

**Table 3:** Logistic regression analysis of variables for spontaneous abortion

Dependent variables	*P* value	ORs	95% CI	
			Lower	Upper
mtDNA copy number	0.196	1.000	1.000	1.001
Maternal age	*0.001*	1.127	1.050	1.210

*ORs*, odds ratio; *CI*, confidence interval; *mtDNA*, mitochondrial DNA The differences in italics were considered significant at P<0.05.

### mtDNA mutations in villous samples

We then asked whether mtDNA of patients in the SA group accumulated more mutations than in the IA group. As suggested by previous research [13], the ratio of mutated-to-whole mtDNA > 70% is often used as a pervasive threshold to predict whether a specific mutation can result in a distinct clinical phenotype. In addition, the pathogenic potential of variants is usually predicted by the CADD score.

**Fig. 2:**
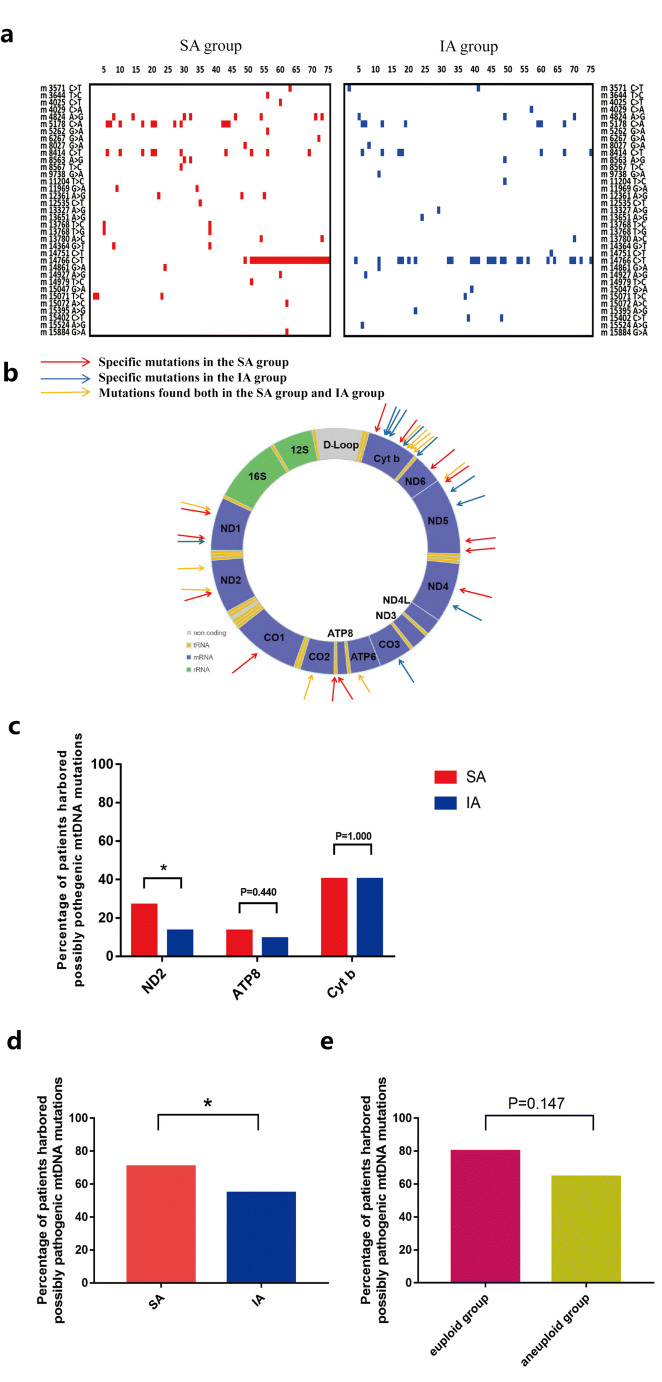
mtDNA mutations in villous samples. **a** Mutation information for each patient. The abscissa represents each patient and the ordinate lists 35 missense mutations with a CADD score > 15 and heteroplasmy ≥ 70%. **b** Circle diagram represents double-stranded mitochondrial DNA. Missense mutations with heteroplasmy > 70% and CADD > 15 found in the placental villus are symbolized by arrows. **c**, **d**, and **e** The percentage of patients harboring possibly pathogenic mtDNA mutations. **c** The percentage of patients with ND2 gene mutation in the SA group was 26.67% (20/75), which was significantly higher than that in the IA group (13.33%, 10/75, *P* = 0.041). However, there was no statistical difference between the SA group (13.33%, 10/75; 40%, 30/75 respectively) and IA groups (9.33%, 7/75; 40%, 30/75 respectively) in the percentage of mutations in ATP8 and Cyt b (*P* = 0.440; *P* = 1.000). **d** Fifty-three of 75 patients in the SA group (70.7%) harbored possibly pathogenic mtDNA mutations, which was significantly higher than in the IA group (54.7%, 41/75; *p* < 0.05). **e** We found no statistical difference between the euploid (80% (24/30)) and aneuploid group (64.4% [29/45], *P* = 0.147. SA, spontaneous abortion; IA, induced abortion; D-Loop, D placement-loop region; Cyt b, cytochrome b, complex III; ND, NADH dehydrogenase, complex I; CO, cytochrome oxidase, complex IV; and ATP, ATPase, complex V

We obtained 35 variants with a CADD > 15 and heteroplasmy ≥ 70% in the SA group and IA group. All of these variants were missense mutations and were primarily found in coding regions (specific mutations are shown in Fig. 2a). With regard to specific point mutations, 14 mutations were only found in the SA group (Fig. 2b, red arrows), 10 mutations were in the IA group (Fig. 2b, blue arrows), and 11 mutations were located in both groups (Fig. 2b, orange arrows).

Moreover, we found a relatively high incidence of mutations in the three mitochondrial genes, ND2, ATP8, and Cyt b. The percentage of patients with ND2 gene mutation in the SA group was 26.67% (20/75), which was significantly higher than that in the IA group (13.33%, 10/75, *P* = 0.041, Fig. 2c). However, we observed no statistical difference between the SA group (13.33%, 10/75; 40%, 30/75 respectively) and IA group (9.33%, 7/75; 40%, 30/75 respectively) in the percentage of mutations in ATP8 and Cyt b (*P* = 0.440; P = 1.000; Fig. 2c).

Interestingly, we found in the SA group, especially in the euploid subgroup that a greater number of patients harbored mtDNA mutations that were possibly pathogenic (70.7%, 53/75; 80%, 53/75 respectively) compared to the IA group (54.7%, 41/75; *P* < 0.05; Fig. 2d). However, we observed no statistical difference between the euploid (80%, 24/30) and aneuploid groups in this endpoint (64.4%, 29/45; *P* = 0.147; Fig. 2e). With respect to the 5 patients in the aneuploid group that had extremely high mtDNA copy numbers, two of them had 0 pathogenic mtDNA mutation, two of them had 1 mtDNA mutation and one had 2 mutations. They had similar mutations compared to other patients. Our results shown above suggest that the accumulation of potentially pathogenic mtDNA mutations was associated with spontaneous abortion, but not with the incidence of euploidy.

## Discussion

During development, the DNA copy number is strictly regulated. Pre-migratory primordial germ cells (PGCs) have less than 10 mitochondria, ovarian PGCs have approximately 100 mitochondria, and there are 200 in oogonia [14]. Then, during oogenesis, the copies act as template for all mtDNA, and the copies are transmitted through the germline and exponentially replicated [15]. The high mtDNA copy number present in the mature metaphase II oocyte is regarded as an energy reserve for subsequent developmental events [8]. However, because there is no later replication of mtDNA except in response to respiratory chain defect [6] or after cryopreservation procedures [7], the mtDNA content of each subsequent, newly formed embryonic cell is reduced as each cell divides [16]. The blastocyst—which contains the first differentiation events of the embryo—initiates mtDNA replication in the trophectodermal cells [16], while the cells of the inner cell mass continue to dilute their mtDNA content as they divide and establish the “mtDNA set point” before gastrulation [17]. The mtDNA set point contains the founder population of mtDNA that contributes to the somatic tissues of the offspring. In the somatic tissues such as heart, muscle, and brain cells, mtDNA is replicated in a cell-specific manner such that the cells give rise to complex functions. Thus, failure to replicate mtDNA prevents embryonic stem cells from completing differentiation [18]. But the embryos can establish compensation mechanism for the respiratory chain defect resulting from high mutation levels, and it was reported that elevated mtDNA copy number was related to aneuploidy and lower implantation potential in women undergoing IVF treatment [19–23]. However, our data did not confirm low or high mtDNA copy numbers in the SA group compared to the IA group, and we observed no statistical difference between the aneuploid and euploid groups. One explanation could be that most of the previous studies used blastomere biopsy or trophectoderm biopsy to evaluate whether an association exists between the mitochondrial DNA copy number with embryo implantation potential or ploidy (some embryos with implantation potential and some without). However, in this paper, chorionic villi were used to evaluate whether an association exists between the mitochondrial DNA copy number with early pregnancy loss or ploidy. All of the embryos possessed implantation potential (with fetal sac). Our results suggested that all of the embryos after implantation have similar mitochondrial copy number, regardless of ploidy, early embryonic loss or not. Our study is therefore the first to prove that early embryonic loss and the incidence of aneuploidy are not related to reduce mtDNA replication. However, it is interesting that five of the samples had very high mtDNA copy number and three of them had dehydrogenase (ND) gene mutation, which was reported to be associated with spontaneous abortions [24, 25]. Maybe the embryos attempted to establish compensation mechanism for the respiratory chain defect resulting from the severe mtDNA mutations.

Rupture of the germinal vesicle, resumption of meiosis, normal fertilization, and a reduction in the incidence of errors during the second meiotic division require high energy consumption [26]. In fact, the mitochondrial DNA (mtDNA) content in oocytes is significantly decreased with advancing maternal age and in patients with low ovarian reserve [27]. Therefore, an energy deficit might be considered to be a cause of chromosomal aberrations and could determine the quality of both oocytes and embryos. Some studies in humans have shown that increasing the oocyte’s mitochondrial mass improves embryo quality in poor prognosis patients [28, 29]. However, a recent RCT study showed that injecting autologous mitochondria into a patient’s own oocyte at the time of ICSI did not benefit the developmental capacity of the treated oocytes, the euploidy status of the embryo and the pregnancy rate (the study was then discontinued) [30]. In summary, the relationship between mtDNA copy number and oocyte quality, embryonic developmental potential, and early embryonic loss still requires further investigation.

The accumulation of mitochondrial DNA (mtDNA) mutations, and the reduction of mtDNA copy number, both disrupt mitochondrial energetics, and may contribute to aging and age-associated phenotypes. However, mtDNA within the female germline constitutes a protected population of mitochondrial genomes that do not harbor all of the variants that can be identified in somatic tissues [31]. With respect to pathogenic mtDNA mutations and deletions that give rise to severe—and sometimes fatal—mitochondrial diseases, it has been demonstrated that the levels of these rearrangements are very different in the germline compared to somatic tissues [32]. This is because the selection or “mitochondrial- bottleneck” events take place during oogenesis so as to refine or select specific variants or mutations that are transmitted through the germline [33]. However, it has been fully confirmed that the female germline still contains variants and mutations that can be transmitted to the offspring [34]. It was reported that the incidence of mitochondrial disease is 1 in 5000 to 1 in 10,000 [34], although 1 in 200 women are carriers of pathogenic rearrangements [35–37]. This clearly indicates there were some selections for and some against these rearrangements.

The combined annotation-dependent depletion (CADD) score has been used to predict pathogenic potential of variants [12] and has been shown to provide better performance than other predictive methods. As recommended by others, a scaled CADD score of 15 was equated with a predisposition to pathogenic mutations [38]. In our study, we counted the number of mutations with a CADD score > 15 and heteroplasmy > 70%, and our data indicated for the first time that more SA patients, especially those with euploid embryos manifested pathogenic mutations compared to IA patients. This suggested that early embryonic loss was one of the mechanisms of selection and purification of pathogenic mtDNA. However, the accumulation of potentially pathogenic mtDNA mutations was not associated with euploidy formation. Whether the accumulation of the mutations caused early embryonic loss requires further investigation. It was reported that mutations in dehydrogenase (ND) genes, mitochondrial tRNA and D-loop of mtDNA in maternal blood may be associated spontaneous abortions [24, 25, 39–41]. It is interesting that we also found the percentage of patients with ND2 gene mutation from villus samples in the SA group was significantly higher than that in the IA group .

Our research has some limitations. First, our samples were derived from placental villous tissues, which may not entirely represent the overall condition of the embryo. Second, our sequencing methods did not detect the loss of large fragment mtDNA. Third, the amplification of mtDNA sequences was based on WGA amplification; it will contain about 15–30% of the nuclear mtDNA homologous pseudo gene sequences (called NUMT) that really is not mtDNA sequence. Lastly, mtDNA copy numbers could be affected by the cellular viabilty and we did not detect it in the villous samples.

In summary, the present study was the first to explore mitochondrial DNA quantity and heteroplasmy in first trimester miscarriages, and we demonstrated that the loss of early embryos and the formation of aneuploidy were not related to the mtDNA copy number. Our research also showed that more patients with spontaneous abortion had possibly pathogenic mtDNA mutations in their chorionic villi, which might help in selecting and purifying pathogenic mtDNA. However, whether the accumulation of these potentially morbific mtDNA mutations caused early embryonic loss requires further investigation.
